# PReS-FINAL-2128: Quality of life and psychosocial aspects in juvenile localized scleroderma (JLS): a cross-sectional study in 40 patients

**DOI:** 10.1186/1546-0096-11-S2-P140

**Published:** 2013-12-05

**Authors:** R Culpo, M Ricca, F Vittadello, G Sequi, F Zulian, G Martini

**Affiliations:** 1 Department of Pediatrics, University of Padua, Padua, Italy

## Introduction

JLS is a chronic, autoimmune disease, characterized by skin and subcutaneous tissues fibrosis, which can extend to underlying tissues up to muscle and bone. JLS can cause a poor quality of life and psychosocial and behavioural problems in affected children particularly when severe deformities such as face asymmetry, joint contractures, and growth disturbances of limbs develop. To date, quality of life and psychological aspects in JLS have been poorly investigated, often with contrasting findings.

## Objectives

To evaluate quality of life and psychosocial aspects of patients with JLS compared with healthy peers and identify specific disease characteristics possibly related to quality of life impairment and psychosocial problems.

## Methods

Two types of questionnaires (Pediatric Quality of Life Inventory 4.0™ Generic Core Scales and Child Behaviour Checklist 6-18/Youth Self Report 11-18) were administered to 40 consecutive patients with JLS aged 6 to 18 years and their parents followed at the Pediatric Rheumatology Unit of Padova. Patients' demographic and clinical data were collected during medical examination and through the review of clinical records. Same questionnaires were administered to a control group of 44 healthy children and their parents.

## Results

In pedsql™ (children forms) no difference was found between JLS group and control group. In pedsql™ (parents forms) children with JLS showed poorer quality of life compared to control group (76.8 vs 84.8, *p = 0.017*), especially in emotional area (64.5 vs 79, *p = 0.004*). In CBCL/6-18 mean scores were lower in activity scale (35.2 vs 41.1, *p = 0,006*) and higher in internalizing problems scale (58 vs 53.2, *p = 0.026*) and depression scale (59.9 vs 55.9, *p = 0.038*) in JLS group compared to control group. In YSR/11-18 mean scores were lower in social competence scale (44.2 vs 49.7, *p = 0.007*) and in total competence scale (40.9 vs 42.4, *p = 0.028*) and higher in internalizing problems scale (54.7 vs 50.9, *p = 0.031*) in JLS group compared to healthy controls. Disease relapses, longer delay in correct diagnosis, onset of disease in adolescence and shorter disease duration significantly correlated with lower quality of life and psychosocial and behavioural problems.

## Conclusion

Our study shows that quality of life is poorer in children with JLS compared to healthy peers. Emotional area and social activities are the most affected ambits and patients show also depressive and internalizing problems. Among patients with JLS, a greater need for psychological support is mainly related to disease relapses, longer diagnostic delay, shorter disease duration and onset in adolescence or pre-adolescence ages. Disease severity in terms of lesion extension or deformities and therapy related issues do not seem related to impairment in the investigated areas.

## Disclosure of interest

None declared.

